# Adjuvant therapy of Chinese herbal medicine for the treatment of adenomyosis: A protocol for systematic review

**DOI:** 10.1097/MD.0000000000020560

**Published:** 2020-06-19

**Authors:** Li Huang, Xiaoli Ji, Xia Wang, Yang Wu, Mei Luo, Xiaotong Hao, Shaobin Wei

**Affiliations:** a Department of Gynecology, Hospital of Chengdu University of Traditional Chinese Medicine, Chengdu; b Department of Gynecology, Hospital of Chongqing Institute of Traditional Chinese Medicine, Chongqing, China.

**Keywords:** adenomyosis, Chinese herbal medicine, Levonorgestrel-releasing intrauterine system, protocol, systematic review

## Abstract

**Background:**

Adenomyosis is benign gynecologic condition with complex etiologies. Common symptoms associated with adenomyosis (AM) include menorrhagia, dysmenorrhea, chronic pelvic pain, metrorrhagia, and dyspareunia. Although Chinese herbal medicine (CHM) has often been utilized for managing AM in clinical practice in China, evidence regarding its efficacy is lacking. This systematic review protocol aims to describe a systematic review to assess the effectiveness and safety of CHM combined with Levonorgestrel-releasing intrauterine system for AM.

**Methods:**

The following 7 databases will be searched from the publishment to December 2019: the Cochrane Central Register of Controlled Trials (CENTRAL), PubMed, EMBASE, China National Knowledge Infrastructure (CNKI), Wanfang Digital Periodicals (WAN FANG), Chinese Biomedical Literature Database (CBM), Chinese Scientific Journal Database (VIP). The primary outcomes will be relief in pain and uterine bleeding. The secondary outcomes include the adverse effects, CA125 variation in peripheral blood, reduction in uterine volume, and endometrial thickness. We will use RevMan V.5.3 to conduct the meta-analysis, if possible. If it is not allowed, a descriptive analysis will be conducted. We will use risk ratio with 95% confidence interval for dichotomous data and the mean difference for continuous data.

**Results:**

This study will provide the latest analysis of the currently available evidence for the efficacy of the adjuvant therapy of CHM for the treatment of AM.

**Registration number:**

OSF (DOI 10.17605/OSF.IO/A2GHY)

**Ethics and dissemination:**

No ethical issues are required. The findings will be published in a peer-reviewed scientific journal.

## 1 Introduction

Adenomyosis is a benign gynecologic condition in which endometrial tissue invades the myometrium, producing a diffusely enlarged uterus. Common symptoms associated with adenomyosis (AM) include menorrhagia, dysmenorrhea, chronic pelvic pain, metrorrhagia and dyspareunia.^[1,2]^ Women with AM are more likely to suffer from depression and infertility.^[3,4]^ A systematic review of six studies shows that AM seems to have an adverse effect on pregnancy outcomes, leading to a higher risk of preterm birth, gestational age, and preeclampsia.^[5]^ As the definite diagnosis of AM could only be based on histological specimens obtained after hysterectomy, the estimated incidence of AM varied between 5% and 70%.^[6]^ About 70% to 80% of AM cases are reported in women in the fortieth and fifth decades. Between 5% and 25% of AM cases are observed in patients younger than 39 years and only 5% to 10% in women older than 60 years.^[6,7]^ Significant progress has been made in imaging techniques such as transvaginal ultrasound and magnetic resonance imaging, providing a conservative diagnostic approach for AM. Despite the high prevalence and the severe symptoms of AM, the pathogenesis of AM is not well understood. Two main theories are proposed in the literature: invagination of the endometrial basalis as a result of activation of the tissue injury and repair mechanism and metaplasia of displaced embryonic pluripotent Mullerian remnants or differentiation of adult stem cells.^[8]^

Pharmacotherapeutic interventions commonly used in the treatment of AM, including oral contraceptives (OCs), gonadotropin-releasing hormone agonist (GnRH-a), progestins, danazol, selective estrogen receptor modulators, selective progesterone receptor modulators, and aromatase inhibitors.^[9]^ Guidelines recommend OCs, Levonorgestrel-releasing intrauterine system (LNG-IUS) and GnRH-a.^[10]^ OCs are first-line treatments that can control dysmenorrhea and reduce recurrence. However, the side effects range from mild nausea, bloating, and altered mood to more severe events such as thromboembolism, cardiac complications, or stroke. GnRH-a causes menopausal symptoms and increases bone loss. LNG-IUS can induce remission of dysmenorrhea and reduce menstrual bleeding, while it often causes side effects of abnormal uterine bleeding and changes in menstrual patterns.

Chinese medical physicians usually use Chinese herbal medicine (CHM) alone or plus Western medicine to treat the symptoms. Several trials have shown the role of CHM in the treatment of AM. The guideline "Guidelines for the diagnosis and treatment of endometriosis” points out that although evidence supporting the efficacy of CHM is uncertain, CHM is used to manage AM in clinical practice. A systematic review evaluated the clinical efficacy and safety of Sanjiezhentong Capsules combined with Levonorgestrel Intrauterine System in the treatment of AM.^[11]^ In this study, the treatment group was set up as Sanjiezhentong Capsule. CHM includes traditional Chinese medicine prescriptions and Chinese patent medicine, whether the evidence is transferable to CHM remains unclear. Another systematic review evaluated the efficacy of CHM versus Western medicine for AM.^[12]^ It searched three Chinese databases, including fourteen clinical trials. The outcome was the total effectiveness rate, which was not clearly described. Although the overall effect seems promising, the treatment details were not proposed due to the lack of subgroup analysis. Therefore, the review had some flaws that threatened the authenticity of their findings.

The present systematic review aims to evaluate the effectiveness and safety of CHM combined with LNG-IUS for AM. This systematic review will provide convincing conclusions by using strict search strategies and objective outcome evaluations.

## 2 Methods

### 2.1 Study registration

This systematic review protocol has been registered as DOI 10.17605/OSF.IO/A2GHY in the Open Science Club (OSF). The protocol is conducted following the Preferred Reporting Items for Systematic Reviews and Meta-Analysis Protocols (PRISMA-P) statement guidelines.^[13]^ And the study will comply with the Preferred Reporting Items for Systematic Reviews and Meta-Analysis (PRISMA) statement guidelines.^[14]^

### 2.2 Criteria for including studies

#### 2.2.1 Types of studies

Any clinical randomized controlled trials (RCTs) related to CHM combined with LNG-IUS for AM without restriction of publication status and languages will be included. The results of completed and ongoing trials published on clinical trial registration platform will also be included. Quasi-randomized controlled studies and small sample studies will be excluded. A small sample study is a study with a sample size of less than 20 patients will be excluded.

#### 2.2.2 Types of participants

Women of reproductive age (18–50 years) diagnosed with AM in terms of either transvaginal ultrasound or magnetic resonance imaging. There were no restrictions on ethnicity, nationality, education, or economic status.

#### 2.2.3 Types of interventions and comparisons

In this review, we will include trials using CHM regardless of frequency or duration. CHM was defined as traditional Chinese herb formula, Chinese patent medicine, and herbal products extracted from Chinese herbs (eg, oral liquid, capsule, tablet, pill, powder, and injection). Studies comparing CHM plus LNG-IUS to LNG-IUS were enrolled. Studies comparing the efficacy of different CHM prescriptions or other complementary and alternative therapeutic interventions will be excluded.

#### 2.2.4 Types of outcome measures

##### 2.2.4.1 Primary outcome measures

The primary outcome measures were:

1. Relief in pain, as assessed by visual analogue scale.
2. Change in menstrual bleeding, as assessed by Pictorial blood assessment chart for menorrhea.

##### 2.2.4.2 Secondary outcome measures

The secondary outcome measures were:

1. Endometrial thickness, as assessed by transvaginal ultrasound.
2. CA125 variation in peripheral blood.
3. Reduction in uterine volume, as assessed by transvaginal ultrasound.
4. Adverse events.

### 2.3 Search strategy

#### 2.3.1 Electronic searches

We will electronically search the following databases from their inception to December 2019: the Cochrane Central Register of Controlled Trials (CENTRAL), PubMed, EMBASE, China National Knowledge Infrastructure (CNKI), Wanfang Digital Periodicals (WAN FANG), Chinese Biomedical Literature Database (CBM), Chinese Scientific Journal Database (VIP). Any clinical RCTs related to CHM combined with LNG-IUS for treating AM without restriction of publication status and languages will be included. The search terms will include: uterine AM, AM, uterine adenomyoma, adenomyoma, adenomyotic, herb, herbal medicine, Chinese herbal medicine, Chinese drug, compound prescription, traditional Chinese medicine, decoction, Chinese formula, Chinese medicine monomer, prescription, Levonorgestrel-releasing intrauterine system, LNG-IUS, Mirena. The search strategies for PubMed are summarized in Table 1. These search terms will be precisely translated for other databases.

**Table 1:**
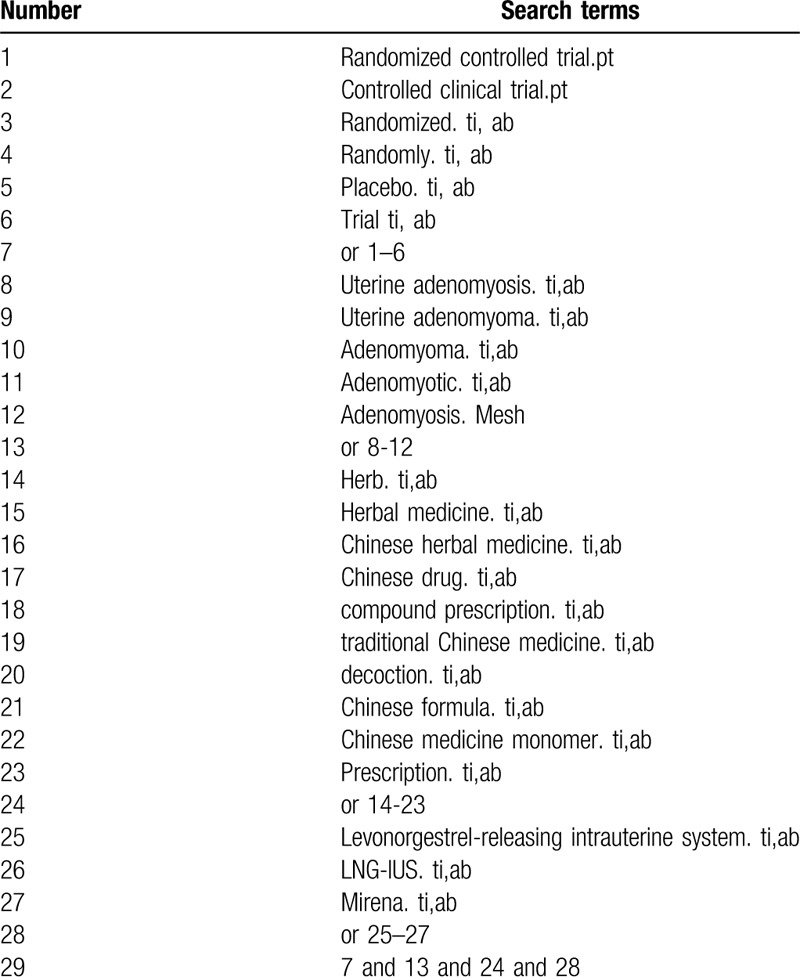
Search strategy for PubMed.

#### 2.3.2 Searching other resources

We will review the reference lists of relevant RCTs and meta-analyses for possible eligible studies. We will also search the ongoing or unpublished trials from the International Clinical Trials Registry Platform (http://www.who.int/ictrp/), the NIH Clinical Trials (https://www.clinicaltrials.gov/), and the Chinese Clinical Register (http://www.chictr.org/).

### 2.4 Data collection and analysis

#### 2.4.1 Selection of studies

**Figure 1:**
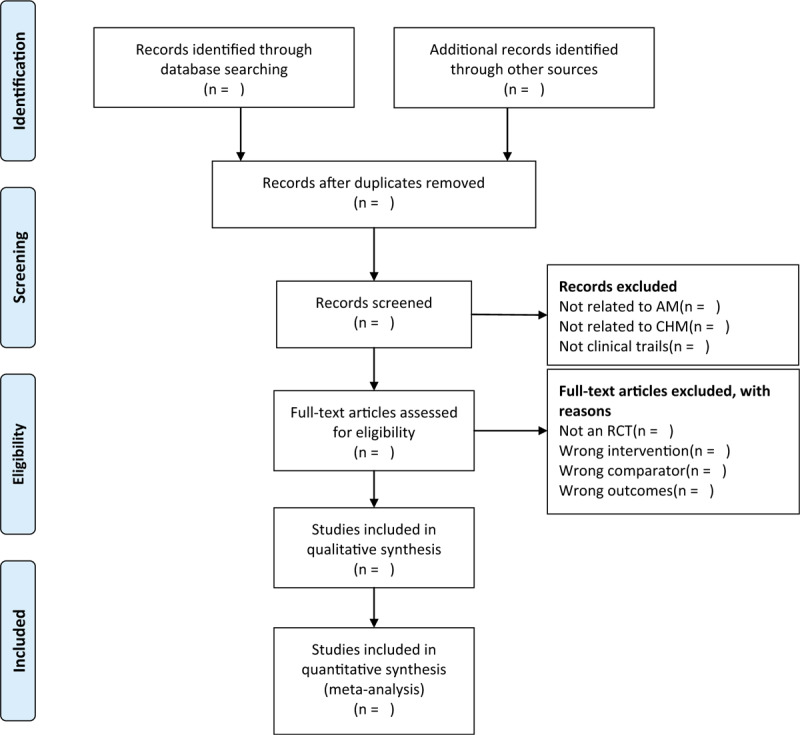
The PRISMA flow diagram of the study selection process.

We will use EndNote software (V.X9.0) to find duplicates and manage records. Two independent reviewers (LH and XW) will check titles and abstracts of studies according to the inclusion criteria. Reviewers (XLJ and YW) will obtain full-text reports for further assessment. Disagreements will be resolved by discussion. If necessary, further argument will be arbitrated by a third reviewer (SBW). The explanations for exclusion will be recorded in an excel data set. The study flow diagram is shown in Figure 1.

#### 2.4.2 Data extraction and management

Two reviewers (ML and XTH) will carefully double check the eligibility of the included studies and extract data using a predesigned data acquisition form. This form will include 4 main domains: general information (title, authors, publication year, country, funding, journal); study methods (design, sample size, method of randomization, allocation concealment, blinding); participant characteristics (age, diagnostic criteria, disease duration); details of intervention (type of CHM, ingredients, treatment duration, treatment frequency); details of comparison (treatment duration); outcomes (primary and secondary outcomes, method of outcome assessments, time points).We will try to contact the corresponding author for unclear information or any missing data.

#### 2.4.3 Assessment of risk of bias in included studies

The methodological quality of each included trial will be evaluated using the Cochrane Collaboration's tool.^[15]^ Two reviewers (XTH and YW) will independently assess the risk of bias. The discrepancy will be discussed to reach an agreement. The third review author (ML) will be consulted, if necessary. Risk of bias assessment categories will include the following 7 domains:

1. randomized sequence generation;
2. allocation concealment;
3. blinding of participants;
4. blinding of outcome assessors;
5. incomplete outcome data;
6. selective outcome reporting;
7. other bias.

We will assign a low, high or unclear risk to each domain. We will try to contact the corresponding author if the basic information is missing about the risk of bias assessment.

#### 2.4.4 Measures of treatment effect

The RevMan 5.3 software will be used for quantitative data synthesis and data analysis. For dichotomous data, such as adverse events, we will use the risk ratio with 95% confidence intervals (Cis) to assess the treatment effect. For continuous data, we will use the mean difference with 95%CIs to evaluate the treatment effect when the same outcome scale is used. We will use the standardized mean difference with 95%CIs when calculating the same outcome variables using different methods.

#### 2.4.5 Dealing with missing data

If there is insufficient or missing data, we will try to contact the corresponding author or the first author. An intention-to-treat analysis that includes all randomized patients will be conducted. We will assume failure for drop-outs in the treatment group and success for drop-outs in the control group.^[16]^ We will use a sensitivity analysis to detect whether the results are inconsistent.

#### 2.4.6 Assessment of heterogeneity

Heterogeneity will be assessed by the *χ*^2^ and *I*^2^ tests. When the *I*^2^ value ≤50%, it is considered to be no heterogeneity between the studies. *I*^2^ value >50% will reveal the presence of substantial heterogeneity in the included studies.^[17]^ Potential clinical heterogeneity will be investigated by subgroup analyses. If *I*^2^ value >75%, meta-analysis will not be performed.^[18]^ And we will qualitatively describe the effectiveness and safety of CHM.

#### 2.4.7 Assessment of reporting biases

If the numbers of studies included in the meta-analysis is sufficient (≥10 studies), we will use funnel plots to detect the reporting bias.^[19]^ An Egger test will be performed to assess the asymmetry of the funnel plot. Since the funnel plot asymmetry does not necessarily imply publication bias, we will try to analyze possible factors such as poor methodological quality and small-sample study.

#### 2.4.8 Data synthesis

Meta-analysis will be performed using RevMan V.5.3. We will synthesize and analyze clinical data according to the level of *I*^2^ value. If *I*^2^ value ≤50%, we will use a fixed effect model. Instead, a random effect model will be used.^[20]^ If *I*^2^ value >75%, we will present a descriptive analysis of each clinical trial.

#### 2.4.9 Subgroup analysis

Subgroup analyses will be conducted to explore the potential causes of heterogeneity if necessary.

We will consider the following subgroup analysis plans.

1. Different types of CHM (Chinese patent medicine, traditional Chinese herbal formula, herbal products extracted from Chinese herbs).
2. Duration of follow-up (1–3 months, up to 6 months).

#### 2.4.10 Sensitivity analysis

To detect the robustness of the main results, we will conduct a sensitivity analysis by removing low-quality trails. In addition, the impact of sample size and missing data will be taken into account.

#### 2.4.11 Grading the quality of evidence

The quality of evidence for all outcomes will be assessed by the Grading of Recommendations Assessment, Development, and Evaluation (GRADE) software. The following areas will be considered: risk of bias, consistency, directness, precision, publication bias and additional points. The quality of evidence will be categorized as high (A), moderate (B), low (C), and very low (D).

## 3 Discussion

According to the theory of TCM, a common pattern underlying these symptoms of AM is the presence of what is known as stagnation of the Qi and blood. Activating blood and resolving stasis therapy is often used alone or plus a series of treatments such as tonifying qi and dispelling dampness therapy to treat the symptoms. The mechanism of CHM treatment for AM may involve improvement of microcirculation, anti-inflammatory activity, and regulation of endocrine and immune systems.^[21–23]^ The mechanism of CHM for AM is not fully understood, more details about its mechanism and the active compounds of CHM remain to be explored.

Many clinical trials have confirmed the effectiveness and safety of CHM in relieving the symptoms of AM. Therefore, we conduct this systematic review and meta-analysis to assess the effectiveness and safety of the adjuvant therapy of CHM for the treatment of for patients with AM. This study has several advantages. The review will synthesize the latest clinical research and evaluate the add-on effect of CHM to LNG-IUS. We will comprehensively summarize and analyze the outcomes, including visual analogue scale score, Pictorial blood assessment chart score, endometrial thickness, serum CA125 level, uterine volume, and adverse events.

There are several potential limitations in the meta-analysis should be concerned. First, different ingredients of each prescription may run the risk of heterogeneity. Second, differences in laboratories and operators also result in heterogeneity. Third, acquiring the complete raw data from original trials is difficult. If we cannot fully obtain the raw data, we will report the details and the results of possible bias. We hope this study will provide the latest analysis of the currently aggregated evidence for the efficacy of the adjuvant therapy of CHM in treating AM, which will benefit patients, practitioners, and healthcare policymakers.

## Author contributions

LH and SBW contributed to the design of the review and coordinated the systematic review. LH and XW applied the search strategy and selection criteria. YW, ML and XTH completed the assessment of the risk of bias. LH and XLJ analyzed the data and wrote this manuscript. SBW critically edited the manuscript. All authors have read and approved the manuscript.

**Conceptualization:** Li Huang, Shaobin Wei.

**Funding acquisition:** Shaobin Wei.

**Methodology:** Li Huang, Xiaoli Ji.

**Resources:** Xia Wang, Yang Wu, Mei Luo, Xiaotong Hao.

**Writing – original draft:** Li Huang, Xiaoli Ji.

**Writing – review & editing:** Shaobin Wei.

## References

[1] Owolabi TO Strickler RC Adenomyosis: a neglected diagnosis. Obstet Gynecol 1977;50:424–7. 904805

[2] Peric H Fraser IS The symptomatology of adenomyosis. Best Pract Res Clin Obstet Gynaecol 2006;20:547–55. 16515888 10.1016/j.bpobgyn.2006.01.006

[3] Taran FA Weaver AL Coddington CC Characteristics indicating adenomyosis coexisting with leiomyomas: a case-control study. Hum Reprod 2010;25:1177–82. 20176591 10.1093/humrep/deq034 PMC2854044

[4] Campo S Campo V Benagiano G Adenomyosis and infertility. Reprod Biomed Online 2012;24:35–46. 22116070 10.1016/j.rbmo.2011.10.003

[5] Razavi M Maleki-Hajiagha A Sepidarkish M Systematic review and meta-analysis of adverse pregnancy outcomes after uterine adenomyosis. Int J Gynaecol Obstet 2019;145:149–57. 30828808 10.1002/ijgo.12799

[6] Azziz R Adenomyosis: current perspectives. Obstet Gynecol Clin North Am 1989;16:221–35. 2664619

[7] Benson RC Sneeden VD Adenomyosis: a reappraisal of symptomatology. Am J Obstet Gynecol 1958;76:1044–57. discussion 57–61. 13583049 10.1016/0002-9378(58)90186-8

[8] Garcia-Solares J Donnez J Donnez O Pathogenesis of uterine adenomyosis: invagination or metaplasia? Fertil Steril 2018;109:371–9. 29566849 10.1016/j.fertnstert.2017.12.030

[9] Vercellini P Vigano P Somigliana E Endometriosis: pathogenesis and treatment. Nat Rev Endocrinol 2014;10:261–75. 24366116 10.1038/nrendo.2013.255

[10] Chinese Medical Association Obstetrics and Gynecology Branch Endometriosis Collaborative Group. Guidelines for the diagnosis and treatment of endometriosis. Chin J Obstet Gynecol 2015;161–9. [Article in Chinese].

[11] Gu ZJ Lu RL Yuan L Meta analysis of the efficacy of San Jie Zhen Tong Capsules combined with Levonorgestrel Intrauterine System in treatment of adenomyosis. China Med Her 2018;15:88–93. [Article in Chinese].

[12] Feng X Shen QB Zhou LJ Meta-analysis of integrated Chinese and Western medicine in the treatment of adenomyosis. Pract Chin West Med Comb Clin 2016;16:45–6. [Article in Chinese].

[13] Shamseer L Moher D Clarke M the PRISMA-P Group. Preferred Reporting Items for Systematic Review and Meta-Analysis Protocols (PRISMA-P) 2015: elaboration and explanation. BMJ 2015;349:g7647. 10.1136/bmj.g7647 25555855

[14] Liberati A Altman DG Tetzlaff J The PRISMA statement for reporting systematic reviews and meta-analyses of studies that evaluate healthcare interventions: explanation and elaboration. BMJ (Clinical research ed) 2009;339:b2700. 10.1136/bmj.b2700 PMC2714672 19622552

[15] Higgins JP Altman DG Gøtzsche PC The Cochrane Collaboration's tool for assessing risk of bias in randomised trials. BMJ 2011;343:d5928 Published 2011 Oct 18. 22008217 10.1136/bmj.d5928 PMC3196245

[16] Aamann L Dam G Rinnov AR Physical exercise for people with cirrhosis. Cochrane Database Syst Rev 2018;12:CD012678. 30575956 10.1002/14651858.CD012678.pub2 PMC6517144

[17] Higgins JPT Thompson SG Quantifying heterogeneity in a meta-analysis. Stat Med 2002;21:1539–58. 12111919 10.1002/sim.1186

[18] Lee S Park J Kim J Acupuncture for postoperative pain in laparoscopic surgery: a systematic review protocol. BMJ Open 2014;4:e006750. 10.1136/bmjopen-2014-006750 PMC4275696 25537788

[19] Higgins JPT, Thomas J, Chandler J, et al. Cochrane Handbook for Systematic Reviews of Interventions version 6.0 (updated July 2019). Cochrane, 2019. Available from www.training.cochrane.org/handbook.

[20] Schilder AGM Chong LY Ftouh S Bilateral versus unilateral hearing aids for bilateral hearing impairment in adults. Cochrane Database Syst Rev 2017;12:CD012665. 29256573 10.1002/14651858.CD012665.pub2 PMC6486194

[21] Yang F Traditional Chinese medicine research and progress in adenomyosis. J China-Japan Friendsh Hosp 2018;32:98–101. [Article in Chinese].

[22] Feng TT Wei SB Wang YH Research on regulatory mechanism of Neiyikangfu Pills on P450arom-COX-2-PGE2 positive feedback loop of mice with adenomyosis. Chin J Tradit Chin Med 2016;31:3250–3. [Article in Chinese].

[23] Wang YH Study on the regulation of estrogen-related receptors ERα, ERβ, GPER, PR-A and PR-B in mice with adenomyosis by the therapy of expelling dampness and dissipating blood stasis. [doctor dissertation]: Chengdu University of Traditional Chinese Medicine; 2015. [Article in Chinese].

